# What about the others: differential diagnosis of COVID-19 in a German emergency department

**DOI:** 10.1186/s12879-021-06663-x

**Published:** 2021-09-17

**Authors:** David Fistera, Annalena Härtl, Dirk Pabst, Randi Manegold, Carola Holzner, Christian Taube, Sebastian Dolff, Benedikt Michael Schaarschmidt, Lale Umutlu, Clemens Kill, Joachim Risse

**Affiliations:** 1 Center of Emergency Medicine, University Hospital Essen, Hufelandstrasse 55, 45147 Essen, Germany; 2 Department of Pulmonary Medicine, University Medicine Essen – Ruhrlandklinik, Essen, Germany; 3 Department of Infectious Diseases, West German Center of Infectious Diseases, University Hospital Essen, University Duisburg-Essen, Essen, Germany; 4 Department of Diagnostic and Interventional Radiology and Neuroradiology, University Hospital Essen, Essen, Germany

**Keywords:** COVID-19, Differential diagnosis, Respiratory infection, Triage, Clinical symptoms, Emergency department, SARS-Cov-2

## Abstract

**Background:**

The ongoing COVID-19 pandemic remains a major challenge for worldwide health care systems and in particular emergency medicine. An early and safe triage in the emergency department (ED) is especially crucial for proper therapy. Clinical symptoms of COVID-19 comprise those of many common diseases; thus, differential diagnosis remains challenging.

**Method:**

We performed a retrospective study of 314 ED patients presenting with conceivable COVID-19 symptoms during the first wave in Germany. All were tested for COVID-19 with SARS-Cov-2-nasopharyngeal swabs. Forty-seven patients were positive. We analyzed the 267 COVID-19 negative patients for their main diagnosis and compared COVID-19 patients with COVID-19 negative respiratory infections for differences in laboratory parameters, symptoms, and vital signs.

**Results:**

Among the 267 COVID-19 negative patients, 42.7% had respiratory, 14.2% had other infectious, and 11.2% had cardiovascular diseases. Further, 9.0% and 6.7% had oncological and gastroenterological diagnoses, respectively. Compared to COVID-19 negative airway infections, COVID-19 patients showed less dyspnea (OR 0.440; *p* = 0.024) but more dysgeusia (OR 7.631; *p* = 0.005). Their hospital stay was significantly longer (9.0 vs. 5.6 days; *p* = 0.014), and their mortality significantly higher (OR 3.979; *p* = 0.014).

**Conclusion:**

For many common ED diagnoses, COVID-19 should be considered a differential diagnosis. COVID-19 cannot be distinguished from COVID-19 negative respiratory infections by clinical signs, symptoms, or laboratory results. When hospitalization is necessary, the clinical course of COVID-19 airway infections seems to be more severe compared to other respiratory infections.

*Trial registration*: German Clinical Trial Registry DRKS, DRKS-ID of the study: DRKS00021675 date of registration: May 8th, 2020, retrospectively registered.

## Background

COVID-19 is an unprecedented situation for society and health care worldwide with more than 211 million cases worldwide and more than 4,400,000 fatalities (Date 08/23/21) [1]. Whereas the majority of infections is mild [2–5] or even asymptomatic [6], about five percent develop a critical disease [5]. Respiratory failure due to severe pneumonia and multiorgan dysfunction with coagulopathy, nephropathy, and affection of the central nervous system cause an estimated case fatality rate at around 0.7 to 2.3% [5, 7, 8]. Laboratory features that have been associated with worse outcome comprise elevation of C-reactive protein, D-dimer, lactate dehydrogenase, acute kidney injury, and troponin [9, 10].

Most patients with mild symptoms can be treated as outpatients, whereas severely ill COVID-19 patients and patients with similar symptoms cross their way in the emergency department (ED). The variety of symptoms is broad and therefore challenging during primary triage, especially to avoid further spread of the infection and to protect staff from infection.

A report of over 370,000 documented symptomatic cases in the U.S. found cough (50%), fever (43%), myalgia (36%), headache (34%), and dyspnea (29%) to be the most common symptoms, but diarrhea (19%), nausea (12%) and taste/smell disorders (< 10%) were also present in a relevant number of cases [11]. Many of these can be found in other common ED diagnoses comprising heart failure, acute coronary syndrome, exacerbation of COPD, and even gastroenterological and oncological diagnoses. Older patients may present with an atypical and therefore misleading clinical picture consisting of falls and malaise [12].

However, a clinical differential diagnosis between COVID-19 and patients presenting with similar symptoms would be very helpful during primary triage.

We, therefore, hypothesized the following:

- Differential diagnosis of COVID-19 is very broad and not limited to respiratory diseases.
- There might be specific differences in symptoms or clinical parameters between COVID-19 positive and negative airway infections that may help to distinguish both entities clinically.
- The clinical course may be more severe in COVID-19 inpatients compared to other infectious airway diseases.

Therefore, we conducted a retrospective analysis of the differential diagnoses of the symptomatic but COVID-19 negative patients in our cohort.

In addition, we tried to identify clinical characteristics and laboratory features that could improve early triage in the ED between patients with proven COVID-19 and patients with acute respiratory infections from other origins as a case–control study.

## Methods

The study was conducted following a similar protocol as a previous one [13], so patients and methods show some overlap.

### Patients

We performed a retrospective, single-center case–control study. Patients with possible symptoms of COVID-19 presenting at the ED of the University Hospital Essen during the first wave of pandemic between March and April 2020 that underwent SARS-CoV2 testing by nasopharyngeal swab and RT-PCR were included in this analysis.

At least one of the following symptoms was mandatory for inclusion: sore throat, dyspnea, cough, chest pain, fever, fatigue, headache, myalgia, nausea, diarrhea, and dysgeusia.

The study was registered at the German Clinical Trials Registry (Trial number: DRKS00021675, Date: May 8, 2020). Our study was approved by the institutional ethics committee, and informed consent was waived (Project number: 20-9310-BO).

Patients and the public were not involved in this study.

### Methods

All patients were tested by a SARS-Cov-2 nasopharyngeal swab (ViroCult®, Medical Wire & Equipment Co. Ltd., Corsham, Wiltshire, UK). For detection of SARS-CoV-2, an reverse transcription polymerase chain reaction (RT-PCR) (SARS-CoV-2 RT-PCR Kit 1.0, Altona Diagnostics GmbH, Hamburg, Germany) was performed [14]. Computed tomography (CT) pulmonary angiography and additional laboratory testing were performed when symptoms of the lower respiratory tract involvement occurred. Retesting or an additional bronchoscopy could be added in case of negative swab testing and ongoing suspicion. Strict isolation measures were kept until COVID-19 was ruled out.

### Parameters

**Fig. 1:**
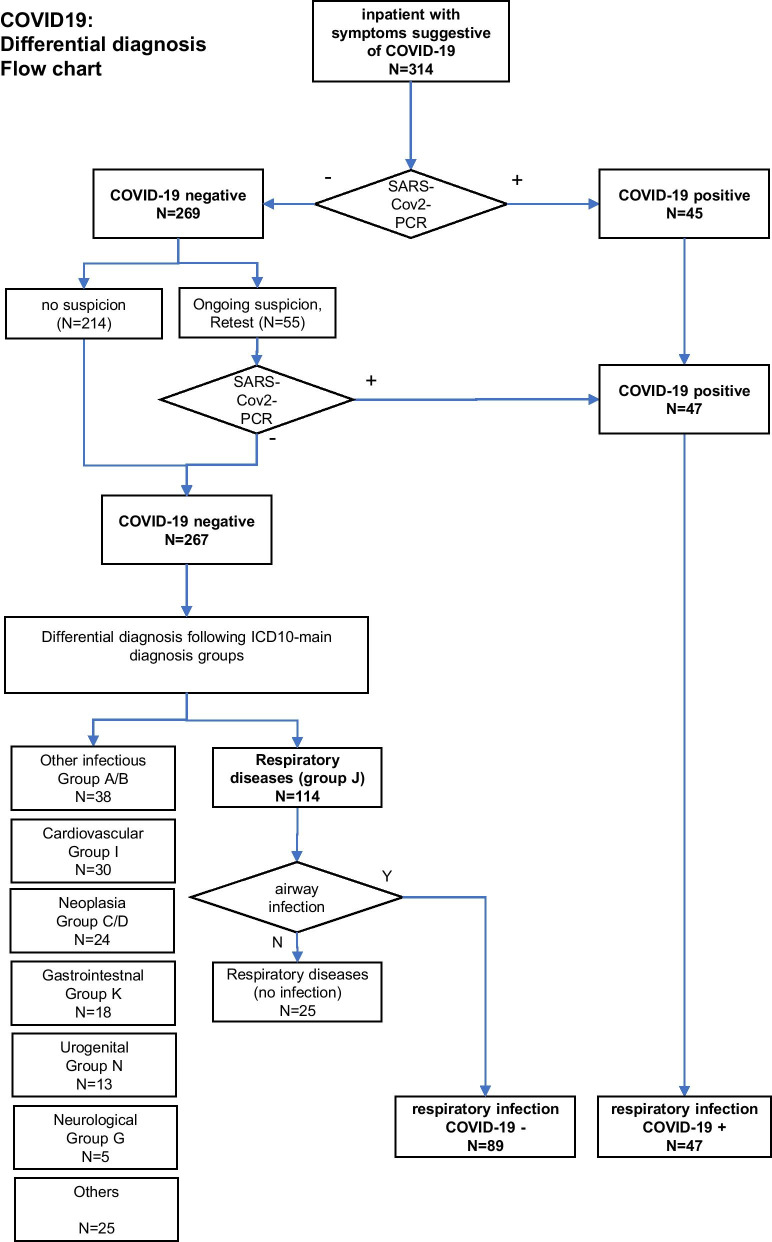
Flow chart of differential diagnosis

We analyzed ICD-10 main diagnosis groups of all symptomatic but COVID-19 negative patients. All patients with acute infectious respiratory diseases were included in the main diagnosis group “J” (respiratory diseases). Those with non-infectious diseases (i.e., pleural effusion, exacerbated COPD without acute infection, etc.) were excluded from further analysis (Fig. 1).

Clinical characteristics, Manchester Triage System (MTS) categories, and laboratory parameters were compared and analyzed between patients with positive swab results for SARS-CoV-2 and those with an acute infectious respiratory disease of other origins, as mentioned above.

Clinical characteristics were symptoms upon ED arrival comprising sore throat, dyspnea, chest pain, cough, fever, fatigue, headache, myalgia, diarrhea, nausea, and dysgeusia.

Laboratory results were white blood cell count, lymphocytes, procalcitonine, C-reactive protein, creatinine, glomerular filtration rate, D-dimers, and troponine.

Patient data were extracted from electronic medical record (ERPath, eHealth-Tec Innovations GmbH, Berlin, Germany; Medico, Cerner Health Services GmbH, Idstein, Germany).

Missing data that could not be extracted from patients’ records were excluded from statistical analyses.

### Statistical analyses

Results were reported as mean ± standard deviations for continuous variables. We used a t-test to evaluate metric data. Data were tested by Levene’s test to assess the equality of variances. For unequal variances, a Welch’s t-test was performed to analyze metric data.

Results for categorical variables were reported as percentages, calculated adjusted odds ratios (ORs), and 95% confidence intervals (CIs) and Pearson’s x^2^ test or the Fisher’s exact test was used. Statistical significance was defined as two-tailed *p* < 0.05. All data were analyzed using SPSS, version 26 (IBM, Armonk, NY, USA).

## Results

**Fig. 2:**
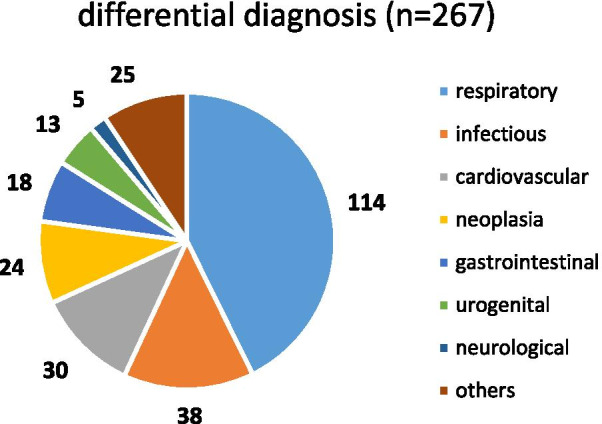
Differential diagnoses of COVID-19 negative patients

A total of 267 SARS-CoV-2 negative patients (61.8% male, mean age 65.6 ± 17.7 years) were analysed for their ICD main diagnosis group. Respiratory diseases (ICD 10 group J) were found in 42.7% (114/267) of cases, followed by 14.2% (38/267) infections of other origins (ICD 10 groups A/B), 11.2% (30/267) cardiovascular (ICD 10 group I), 9.0% (24/267) oncological (ICD 10 groups C/D), 6.7% (18/267) gastrointestinal (ICD 10 group K), 4.9% (13/267) urogenital (ICD 10 group N), 1.9% (5/267) neurological (ICD 10 group G), and 9.4% (25/267) miscellaneous diseases (all remaining ICD 10 groups; Fig. 2).

Further evaluation of the respiratory diseases group (n = 114) resulted in the exclusion of 25 cases of non-infectious respiratory diseases (pulmonary edema, non-infectious exacerbation of COPD, pleural effusion, asthma, and hypercapnic respiratory failure), so a total of 89 COVID-19 negative respiratory infections (50 pneumonia (J18.0-J18.9), 17 influenza/viral pneumonia (J10.0, J10.1, J10.8, J12.1, J12.8), six upper respiratory tract infections (J06.8, J06.9), 16 acute bronchitis (J20.9, J22, J44.01, J44.09)) were included.

A total of 136 patients [mean age: 68 years ± 17.5 year; 46 female (33.8%)] were included in the analysis. Baseline characteristics are summarized in Table 1*.* According to the MTS, 14 patients were classified as “red” (10.3%), 12 patients as “orange” (8.8%), 50 patients as “yellow” (36.8%), 58 patients as “green” (42.6%), and two patients as “blue” (1.5%).

**Table 1:** Characteristics of inpatients with airway infections

	All (n = 136)	COVID19+ (n = 47)	COVID19– (n = 89)	Odds ratio (95% confidence interval)	*p-*value
Age, mean (± SD, range)	68 (± 17.54, 23–97)	70 (± 17.65, 23–94)	67 (± 17.51, 24–97)		0.420
Male gender, n (%)	90 (66.2)	31 (66.0)	59 (66.3)	0.985 (0.481–2.141)	0.969
Manchester triage, n (%)					
Red	14 (10.3)	4 (8.5)	10 (11.2)	0.735 (0.217–2.483)	0.619
Orange	12 (8.8)	3 (6.4)	9 (10.1)	0.606 (0.156–2.355)	0.466
Yellow	50 (36.8)	15 (31.9)	35 (39.3)	0.723 (0.343–1.525)	0.394
Green	58 (42.6)	25 (53.2)	33 (37.1)	1.928 (0.942–3.948)	0.071
Blue	2 (1.5)	0 (0)	2 (2.2)	0.987 (0.947–1.009)	0.301
Medical history, positive for, n (%)					
Cardiac	85 (62.5)	29 (61.7)	56 (62.9)	0.904 (0.436–1.877)	0.769
Pulmonary	44 (32.4)	10 (21.3)	34 (38.2)	0.429 (0.189–0.973)	0.108
PE/thrombosis	9 (6.6)	2 (4.3)	7 (7.9)	0.53 (0.106–2.673)	0.437
Renal	**26 (2.9)**	**4 (8.5)**	**22 (24.7)**	**0.283 (0.091–0.879)**	**0.025**
Cancer	31 (22.8)	10 (21.3)	21 (23.6)	0.87 (0.373–2.053)	0.808
Smoker, n (%)					
Never	26 (19.1)	9 (19.1)	17 (19.1)	0.997 (0.406–2.448)	0.995
Yes	**16 (11.8)**	**1 (2.1)**	**15 (16.9)**	**0.107 (0.014–0.839)**	**0.011**
Quitted	9 (6.6)	4 (8.5)	5 (5.6)	0.640 (0.163–2.506)	0.519
Unknown	85 (62.5)	33 (70.2)	52 (58.4)	0.596 (0.281–1.267)	0.177

Bold values mark a significant parameter (*p* < 0,05) and were used to improve readibility and to emphasize main results

Of all COVID-19 patients, 40% (19/47) reported dyspnea, while this clinical feature was present in 61% (54/89) of non-COVID-19 patients (OR 0.440; *p* = 0.024). Among the COVID-19 patients, 15% reported taste disorders (7/47), whereas only 2% (2/89) of the COVID-19 negative patients did so (OR 7.631; *p* = 0.005). Significant differences between the two groups were not observed for other clinical features or vital parameters (Table 2).

**Table 2:** Group comparison COVID-19 versus COVID-19 negative airway infections

	All (n = 136)	COVID19+ (n = 47)	COVID19– (n = 89)	Odds ratio (95% confidence interval)	*p*-value
Symptoms, n (%)					
Dyspnoe	**73 (53.7)**	**19 (40.4)**	**54 (60.7)**	**0.440 (0.214–0.905)**	**0.024**
Sore throat	10 (7.4)	4 (8.5)	6 (6.7)	1.287 (0.345–4.806)	0.707
Cough	79 (58.0)	25 (28.1)	54 (60.7)	0.737 (0.361–1.503)	0.400
Fever	85 (62.5)	31 (66.0)	54 (60.7)	1.256 (0.600–2.627)	0.545
Headache	14 (10.3)	5 (10.6)	9 (10.1)	1.058 (0.333–3.360)	0.924
Fatigue	68 (50.0)	22 (46.8)	46 (51.7)	0.823 (0.405–1.670)	0.589
Myalgia	24 (17.6)	6 (12.8)	18 (20.2)	0.577 (0.212–1.570)	0.278
Chest pain	11 (8.1)	2 (4.3)	9 (10.1)	0.395 (0.82–1.909)	0.234
Nausea	22 (16.2)	6 (12.8)	16 (18.0)	0.668 (0.242–1.839)	0.433
Diarrhea	35 (26.5)	13 (27.7)	22 (24.7)	1.164 (0.523–2.592)	0.709
Dysgeusia	**9 (6.6)**	**7 (14.9)**	**2 (2.2)**	**7.631 (1.513–38.292)**	**0.005**
Death, n (%)	**14 (10.3)**	**9 (19.1)**	**5 (5.6)**	**3.979 (1.242–12.673)**	**0.014**
Treatment, n (%)					
O_2_-therapy	52 (38.2)	20 (42.6)	32 (36.0)	1.319 (0.641–2.717)	0.451
Ventilator	2 (1.4)	0 (0.0)	2 (2.2)	0.978 (0.947–1.009)	0.137
Intensive care	24 (17.6)	6 (12.8)	18 (20.2)	0.577 (0.212–1.570)	0.278
Intermediate care	12 (8.8)	4 (8.5)	8 (9.0)	0.942 (0.268–3.307)	0.926
Time of admission (days)	**6.8 (± 6.4)**	**9.0 (± 8.1)**	**5.6 (± 5.0)**		**0.014**
Vital parameters					
Respiratory rate/min (± SD)	37.2 (± 1.2)	23.7 (± 7.4)	22.3 (± 6.7)		0.283
Heart rate/min (± SD)	96.7 (± 21.6)	93.7 (± 16.6)	98.4 (± 23.8)		0.235
Saturation, O_2_ in % (± SD)	94.3 (± 7.1)	94.9 (± 4.0)	94.0 (± 8.4)		0.456
Temperature in °C, (± SD)	37.2 (± 1.2)	37.3 (± 1.0)	37.2 (± 1.2)		0.552
BP systolic in mmHg (± SD)	132.5 (± 25.6)	136.2 (± 24.1)	130.52 (± 26.3)		0.219
BP diastolic in mmHg (± SD)	79.0 (± 17.4)	82.9 (± 18.1)	77.0 (± 16.7)		0.057
Laboratory values					
C-reactive proteine, mg/L	9.57 (± 7.86)	8.2 (± 5.8)	10.3 (± 8.7)		0.100
Procalcitonine, µg/L (± SD)	4.53 (± 36.75)	0.42 (± 1.49)	6.74 (± 45.47)		0.354
Troponin I, µg/L (± SD)	73.27 (± 268.64)	76.59 (± 278.7)	71.33 (± 265.0)		0.928
LDH, U/L (± SD)	**370.67(± 248.34)**	**439.5 (± 264.9)**	**335.8 (± 233.4)**		**0.025**
Creatinine, mg/dL (± SD)	1.21 (± 0.87)	1.18 (± 0.90)	1.23 (± 0.86)		0.729
GFR, mL/min (± SD)	58.10 (± 19.10)	58.7 (± 19.18)	57.8 (± 19.17)		0.805
D-dimer, mg/L (± SD)	3.10 (± 6.13)	4.29 (± 7.95)	2.44 (± 0.61)		0.222
WBC/mm^3^ (± SD)	12.04 (± 21.44)	8.00 (± 4.11)	14.1 (± 26.04)		0.115
Lymphocytes/mm^3^ (± SD)	2.39 (± 10.99)	1.20 (± 1.40)	3.1 (± 13.95)		0.322

Bold values mark a significant parameter (*p* < 0,05) and were used to improve readibility and to emphasize main results

Except for renal disorders (8.5% vs. 24,7%; OR 0.283; p = 0.025), no significant differences were observed for clinical preconditions (cardiac or pulmonary disorders, previous thrombosis or pulmonary embolism, and oncological diseases) between COVID-19 positive and COVID-19 negative patients. The number of active smokers was significantly higher in the COVID-19 negative group (16.9% vs. 2.1%; OR 0.107; *p* = 0.011). However, there was a high number of patients with unknown smoking status in both groups (70.2% vs. 58.4%; *p* = 0.177).

COVID-19 inpatients had a significantly higher mortality than the group with pulmonary infections from other origin (19.1% versus 5.6%; OR 3.979; *p* = 0.014).

The duration of hospital stay was longer among COVID-19 patients (9.0 vs. 5.6 days; *p* = 0.014) than COVID-19 negative patients.

In the group of COVID-19 patients, mean levels of lactate dehydrogenase (LDH) were significantly higher (439.5 vs. 335.8 U/L; *p* = 0.025). The mean procalcitonine levels tended to be higher in COVID-19 negative patients (6.74 vs. 0.42 µg/L) but were not significantly different (*p* = 0.354).

All remaining laboratory values, vital parameters, and treatment modes were similar between the two groups (Table 2).

Of the initially 269 SARS-CoV-2 negative patients, 55 had been retested for ongoing clinical suspicion of COVID-19, 14 of these by PCR from bronchoalveolar fluid (BAL). Two of the retested ones revealed to be positive during retesting.

## Discussion

Early triage and differential diagnosis of patients presenting with typical clinical symptoms of COVID-19 remain very challenging but relevant. Our study had the following main findings:

1. Differential diagnosis of typical COVID-19 symptoms is very broad and comprises many common respiratory, infectious, and cardiovascular diseases, whereas respiratory diseases are the most frequent. Diseases from nearly every field of clinical medicine can mimic a clinical picture like that of COVID-19, with respiratory diseases being the most prevalent. Older patients may be even more challenging since the clinical picture may be atypical with syncope and malaise [12].
2. Patients with COVID-19 present with similar symptoms as COVID-19 negative respiratory infections, so clinical discrimination is not reliable. Dyspnea is less frequently found in our COVID-19 patients, whereas dysgeusia is significantly more prevalent. The latter finding has been described by other studies before and can be found in up to 44% of cases following meta-analyses [15]. Whenever present, dysgeusia should raise high suspicion for COVID-19, especially during a pandemic. Dyspnea is a typical symptom of COVID-19, which could be found in about 29% of cases in a study of 270,000 patients in the U.S. [11]. Controversially, several authors described a specific phenomenon called “happy hypoxemia” in COVID-19 with a disconnect between the severity of hypoxemia and relatively mild respiratory discomfort [16, 17]. Therefore, dyspnea might be less frequent in our COVID-19 positive patients than in other respiratory infections. Elevated levels of LDH have been described before [18] and were significantly higher among non-survivors in a case series from Wuhan [10] so this finding in our COVID-19 patients is in line with the more severe clinical course of this group. The tendency towards higher procalcitonine levels in COVID-19 negative patients may be explained by a higher rate of bacterial infections, such as pneumonia, since elevated procalcitonine levels can usually only be found in advanced, respectively complicated courses of COVID-19 [4]. Case numbers might have been too small to reach significance here. The significantly lower frequency of smokers in the COVID-19 group should be interpreted cautiously since the rate of unknown smoking status is 70%, thwarting the attempt to draw any further conclusions. Therefore, no clinical sign or symptom nor any of the analyzed laboratory values will be able to predict COVID-19 status in a reliable way. However, dysgeusia, when present, should raise a high suspicion of COVID-19 during a pandemic. A strict isolation policy and frequent SARS-CoV-2 testing will remain the most important measures to keep control of the situation.
3. When inpatient treatment for respiratory infections is needed, COVID-19 patients seem to take a more severe clinical course. The mortality of our COVID-19 positive inpatient patients is significantly higher than in the COVID-19 negative group. The mortality rate of 19.1% is comparable to those found by Petrilli et al. [19], who reported mortality of 24.1% among inpatients in New York City. The COVID negative group is a heterogeneous one, comprising different kinds of respiratory infections with pneumonia as the most frequent diagnosis (50/89). Inpatients with CAP showed 30-day mortality of 11.9% in Europe in one study [20], so the lower mortality of the COVID negative group might be explainable hereby. This finding is supported by a study that described a more severe course of COVID-19 than Influenza during the first wave in the Toronto area [21]. A more severe course of the disease can also explain the significantly longer length of hospital stay (9.0 vs. 5.6 days; *p* = 0.014) among the COVID-19 positive patients in our study. Notably, bacterial or fungal superinfections seem to occur seldom in the course of COVID-19 [22].
4. The false-negative rate of nasopharyngeal swab testing was low, and 55 patients were retested due to ongoing clinical suspicion of COVID-19, some even more than one time including bronchoalveolar specimens in 14 cases. Only two more positive cases (3.6%) could be found, both by BAL. This suggests that the false-negative rate is low whenever experienced and well-trained staff carries out a nasopharyngeal SARS-Cov2 swab. Previous research has reported rates of 11% for false-negative PCR results in COVID-19 [23]. Of note, all patients were symptomatic, so those in the very early stages of disease who might carry a higher likelihood of false-negative testing were scarce in our study.

### Limitations

Due to retrospective data collection, selection bias and errors in data entry could not be completely excluded.

A standardized form on symptoms was established in the electronic record; however, completeness of medical history and clinical complaints cannot be guaranteed. This study is a single-center study, and for this reason, data should not be generalized. Since the first wave of COVID-19 had been controlled early in Germany, the sample size is limited.

Further, we included only patients admitted to our ED, whereas the number of outpatient treatment may be higher in COVID-19 than in non-COVID-19 respiratory infections.

In our cohort, the number of patients with unknown smoking status is very high (62.5%). Therefore, influence of smoking on outcome should be interpreted very cautious.

## Conclusions

Differential diagnoses of COVID-19 are plentiful and comprise many common diseases, most notably ailments associated with respiratory impairment. Triage remains challenging in the emergency department since there are no reliable clinical or laboratory parameters to distinguish safely between COVID-19 and airway infections of other origins. When inpatient, COVID-19 takes a more severe clinical course than comparable COVID-19 negative airway infections. Therefore, a strict isolation policy together with broad and rapid testing will remain the most important measures for the months to come.

## Data Availability

The anonymized dataset supporting these conclusions is available upon reasonable request from the corresponding author.

## Abbreviations

BAL: Bronchoalveolar lavage
CAP: Community-acquired pneumonia
COPD: Chronic obstructive pulmonary disease
COVID-19: Coronavirus disease subtype 2019
CT: Computed tomography
ED: Emergency department
ICD: International code (of) diagnosis
MTS: Manchester triage scale
NSTEMI: Non-ST segment elevation myocardial infarction
PCR: Polymerase chain reaction
SARS-Cov2: Severe acute respiratory syndrome coronavirus 2
